# C3 Glomerulonephritis Presenting With Nephritic and Nephrotic Syndromes: Spontaneous Remission After Six Months on Dialysis

**DOI:** 10.7759/cureus.50396

**Published:** 2023-12-12

**Authors:** Francisco Gonçalves, Nídia Marques, Roberto Silva, Luis Mendonça, Bernardo Faria

**Affiliations:** 1 Nephrology, Centro Hospitalar Universitário de São João, Porto, PRT; 2 Anatomical Pathology, Centro Hospitalar Universitário de São João, Porto, PRT

**Keywords:** kidney biopsy, recovery of normal renal function, hemodialysis, cfhr, c3 glomerulopathy

## Abstract

C3 glomerulopathy is a rare and complex renal disease driven by complement dysregulation, with variable presentation and pathophysiology. We report the case of a middle-aged male patient presenting with nephritic and nephrotic syndromes and low serum C3, whose biopsy established the diagnosis of C3 glomerulonephritis. He was found to be homozygous for the complement factor H-related protein (CFHR)3-CFHR1 deletion, which has been associated with the development of anti-factor H autoantibodies. However, the lack of consistent and accessible nephritic factor assays prevented full clarification of the mechanisms involved in the disease. Interestingly, despite not receiving treatment due to suspicion of malignancy and perceived poor renal prognosis, there was spontaneous recovery after six months on hemodialysis. This case reflects the enduring challenges in establishing the diagnosis and prognosis of C3 glomerulonephritis.

## Introduction

C3 glomerulopathy (C3G) is driven by dysregulation of the alternative complement pathway and comprises both C3 glomerulonephritis (C3GN) and dense deposit disease [1]. Excessive activation of the alternative complement pathway may be due to the presence of autoantibodies or, less frequently, mutations in the regulatory proteins of the alternative complement pathway [2].

Clinical presentation is heterogeneous, with variable degrees of renal dysfunction, hypertension, hematuria, and proteinuria, often in the nephrotic range; nephrotic syndrome is present in up to 50% of cases [2]. Serum C3 levels are low in most patients and are often the first clue for diagnosis [1]. Most biopsies reveal a membranoproliferative pattern but, independently of light microscopy findings, diagnosis of C3G is based on the predominance of C3 deposition over immunoglobulin deposits in immunofluorescence [3].

Immunosuppression, namely, with glucocorticoids and mycophenolate mofetil, and terminal complement pathway blockers have proven useful in a subset of patients [1]. However, neither universally effective nor disease-specific treatments are available, and there is progression to end-stage kidney disease in up to half of adult C3G patients.

## Case presentation

A 48-year-old man was admitted for dyspnea and anasarca. He was a former smoker with arterial hypertension under control, treated dyslipidemia, and alcoholic Child-A cirrhosis. There was no fever or history of other infection-related symptoms and no new drugs had been started. His family history was negative for kidney disease.

Initial investigations revealed Kidney Disease Improving Global Outcomes stage 3 acute kidney injury and hypoalbuminemia, with no thrombocytopenia or features of microangiopathic hemolytic anemia. Urinalysis revealed dysmorphic hematuria and daily protein excretion of 6.4 g. Renal ultrasound was unremarkable. The presence of nephritic and nephrotic syndromes was established, and a kidney biopsy was performed.

**Figure 1:**
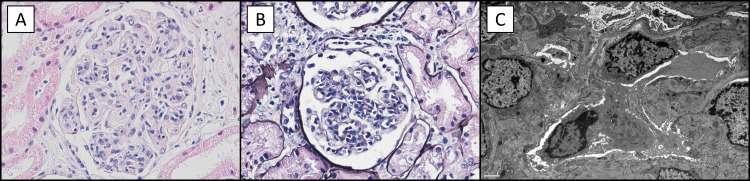
Kidney biopsy. (A) Mesangial and endocapillary hypercellularity with glomerular tuft lobulation (hematoxylin-eosin, 400×). (B) Thickened capillary walls with double contours (Jones methenamine silver, 400×). (C) Diffuse effacement of the pedicels, mesangial proliferation, and deposition of immune complexes in the mesangial, subendothelial, and intramembranous areas (electron microscopy).

Optical microscopy revealed mesangial and endocapillary hypercellularity with glomerular tuft lobulation (Figure 1A) with thickened capillary walls and double contours (Figure 1B). There was predominance of C3 (+++) over IgG (+), IgA (+), lappa (+) and lambda (+) in immunofluorescence. Electron microscopy unveiled diffuse podocyte foot process effacement, mesangial proliferation, and deposition of immune complexes in the mesangial, subendothelial, and intramembranous compartments (Figure 1C). There were no signs of acute tubular necrosis, interstitial fibrosis, or significant vascular lesions. The histological diagnosis was membranoproliferative glomerulonephritis with C3 dominant staining, compatible with C3GN on electron microscopy.

C3 serum levels were persistently low, supporting the diagnosis of C3GN. Workup for autoimmune disease, monoclonal gammopathy, and infection, including spontaneous bacterial peritonitis, was negative. However, computed tomography raised suspicion of peritoneal carcinomatosis.

Hemodialysis was started due to oliguria and worsening renal function. As the patient was dialysis dependent and malignancy was being considered, no immunosuppression was given.

A genetic study of complement regulatory proteins identified a deletion of complement factor H-related protein (CFHR)3-CFHR1 in homozygosity. The presence of anti-factor H (CFH) and anti-factor B antibodies was ruled out, but only three months after presentation. Measurement of C3 nephritic factor (C3NeF) was not available.

On follow-up, ascites cytology and peritoneal biopsy were negative for neoplasia. Surprisingly, spontaneous recovery of renal function was documented six months post-diagnosis. At one year of follow-up, the patient had preserved kidney function, no hematuria, no proteinuria, and C3 levels were normal.

## Discussion

Spontaneous remission is compatible with infection-related glomerulonephritis, although recovery generally occurs between eight and twelve weeks from diagnosis. Furthermore, there was no clear history of infection before presentation and active infection was ruled out. Unfortunately, specific markers of infection-associated glomerulonephritis are not available [2].

The patient was homozygous for a CFHR3-CFHR1 deletion. This is the most common structural change in the CFH gene cluster and is associated with the development of anti-CFH autoantibodies [4]. Anti-CFH antibodies are present in 20% of atypical hemolytic-uremic syndrome (aHUS) cases [5], and, in total, 80% of aHUS patients who develop anti-FH autoantibodies are homozygous for this variant [6]. This patient had no features of thrombotic microangiopathy, either systemic or on kidney biopsy. However, anti-CFH antibodies have originally been described in C3G and are present in 10% of patients with this diagnosis [7]. It is hypothesized that they can trigger aHUS or, less commonly, C3G depending on the specific region of CFH they target [7,8]. As testing for anti-CFH antibodies was performed only later in the disease course, its presence would be a possible link between homozygosity for the CFHR3-CFHR1 deletion and the C3G phenotype.

Likewise, lack of information regarding C3NeF is a hindrance to the C3GN workup performed, as these autoantibodies are present in up to 50% of C3GN. Still, accurate analysis of C3NeF is challenging and not available in most centers due to the heterogenic nature of these autoantibodies, differences in properdin dependence, and inconsistent results from different assays [9]. Other nephritic factors, whose presence was not dismissed, have been described, such as C5 nephritic factors and, less commonly, C4 nephritic factors and factor B autoantibodies [1].

Complete spontaneous recovery was documented after six months. Caravaca-Fontán et al. [10] found that the main predictors of renal failure in C3GN were the degree of renal dysfunction, degree of proteinuria, and total chronicity on biopsy. This patient would be classified as having a high risk for renal failure on the described nomogram. Still, that would predict a median renal survival of 20 months. Aside from the absence of chronicity lesions on renal biopsy (and therefore potential for recovery), the reason for resolution is unclear.

## Conclusions

Clarifying the dominant pathogenic mechanisms in C3G can prove taxing due to the lack of availability and standardization of comprehensive autoantibody assays and genetic tests. This, in turn, curtails proper tailoring of therapy and follow-up. This report exemplifies these enduring challenges and describes spontaneous recovery after several months of dialysis dependency, which should prompt continual monitoring after C3G diagnosis.
